# Immune cell ratio and coagulation markers in assessing prognosis of asthma: a cross-sectional study from Saudi Arabia

**DOI:** 10.3389/fimmu.2023.1206636

**Published:** 2023-07-17

**Authors:** Fahad M. Aldakheel, Zamil A. Alruwaili, Shatha A. Alduraywish, Amal F. Alshammary, Ayesha Mateen, Rabbani Syed, James John

**Affiliations:** ^1^ Department of Clinical Laboratory Sciences, College of Applied Medical Sciences, King Saud University, Riyadh, Saudi Arabia; ^2^ Prince Sattam Chair for Epidemiology and Public Health Research, Department of Family and Community Medicine, College of Medicine, King Saud University, Riyadh, Saudi Arabia; ^3^ Department of Family and Community Medicine, College of Medicine, King Saud University, Riyadh, Saudi Arabia; ^4^ Department of Pharmaceutics, College of Pharmacy, King Saud University, Riyadh, Saudi Arabia; ^5^ Department of Medical Laboratory Technology, School of Allied Health Science, Sathyabama Institute of Science and Technology, Chennai, India

**Keywords:** asthma, coagulation, d-dimer, immune cell ratio, Saudi Arabia

## Abstract

Asthma affects a significant number of individuals in Saudi Arabia, with increasing prevalence worldwide, leading to a considerable impact on their quality of life and frequent hospitalizations. In this study, we aimed to explore the relationship between the immune cell ratio and coagulation markers, specifically to identify the occurrence of coagulation abnormalities associated with asthma. To achieve this, we assessed asthma history and severity using a questionnaire while analyzing coagulation biomarkers through venous blood samples. The biomarkers examined included d-dimer, prothrombin time (PT), partial thromboplastin time (PTT), and the international normalized ratio (INR). In addition, we evaluated various hematological parameters such as blood cell counts and hemoglobin (HGB) levels. Our findings revealed compelling evidence, showing significantly elevated levels of d-dimer and the eosinophil-to-neutrophil (ENR) ratio in asthma cases compared to the controls. Moreover, we observed a positive correlation between d-dimer levels and the ENR, with each unit increase in d-dimer associated with a 0.0006 increase in the ENR among asthma cases. These results highlight the potential of assessing ENR and d-dimer levels as predictive indicators for disease prognosis and the development of coagulation abnormalities in individuals with asthma. By shedding light on the relationship between immune cell ratios and coagulation markers in the context of asthma, our study contributes to a better understanding of disease progression and the associated complications. These insights can potentially lead to improved management strategies and better outcomes for asthma patients.

## Introduction

Asthma is characterized by chronic airway inflammation, resulting in symptoms such as chest tightness, wheezing, and coughing (1, 2). It is a heterogeneous disease that exhibits not only hyper-responsiveness but also persistent and irreversible airflow limitation due to airway remodeling (3, 4). Studies have associated airway wall remodeling with the fibronectin-induced proliferation of epithelial cells and airway smooth muscles (3–5). Patients with chronic asthma often experience a progressive decline in lung function and may develop steroid resistance, necessitating the development of new therapeutic strategies targeting inflammation (6). Asthma has also been associated with an increased risk of pulmonary embolism (PE) and deep vein thrombosis (DVT), indicating a systemic inflammatory process with prothrombotic effects (7, 8). In Saudi Arabia, studies have reported high rates of wheezing and physician-diagnosed asthma among adults and adolescents (9–11).

Immune cells, specifically eosinophils and neutrophils, play a crucial role in the inflammatory response associated with asthma. Eosinophils are often involved in allergic and eosinophilic asthma, while neutrophils are associated with non-allergic and neutrophilic asthma. These cells contribute to airway inflammation, bronchial hyperresponsiveness, and tissue damage in the lungs, thereby influencing disease severity and prognosis. Recent research has compared eosinophilia and the neutrophil–lymphocyte ratio (NLR) in hospitalized and non-hospitalized children with asthma, revealing a prothrombotic state in severe asthmatics (12). Eosinophils play a significant role in the development of both allergenic and non-allergenic asthma, while neutrophilic airway inflammation is associated with severe disease stages triggered by Th1 lymphocytes (13, 14).

Coagulation markers, on the other hand, have emerged as potential contributors to asthma pathogenesis. Studies have shown that asthma is not solely limited to airway inflammation but also involves systemic inflammatory processes. The prothrombotic effects of asthma have been observed, indicating an association between inflammation and coagulation abnormalities. Abnormalities in coagulation markers, such as elevated levels of d-dimer, prothrombin time, partial thromboplastin time, and the international normalized ratio, have been reported in patients with asthma and may contribute to the increased risk of thrombotic events. Local coagulation activation in asthma has been demonstrated through the detection of high concentrations of thrombin and its complex with antithrombin in sputum and bronchoalveolar lavage fluid after allergen exposure, highlighting the existence of coagulation abnormalities in the pulmonary compartment (15, 16). Case-control studies have documented the presence of a prothrombotic state among severe asthmatics, with higher levels of hemostatic markers such as endogenous thrombin potential (ETP), plasmin-α2-antiplasmin complex, and von Willebrand factor correlating with increasing disease severity (17). The protease-activated receptors have been identified as molecular links between coagulation and allergic inflammation in asthma (2).

From earlier study, it is very much clear that there is a relationship between immune cell ratios (specifically, the eosinophil-to-neutrophil ratio) and coagulation markers in assessing the prognosis of asthma. Higher immune cell ratios are associated with an increased risk of coagulation abnormalities and poor prognosis in asthma. Therefore, by investigating the relationship between immune cell ratios (specifically, the eosinophil-to-neutrophil ratio) and coagulation markers, the authors aim to gain insights into the interplay between inflammation and coagulation in asthma. Understanding the role of these markers in asthma could provide valuable information for predicting disease prognosis, identifying coagulation abnormalities, and potentially developing targeted therapeutic strategies to improve patient outcomes.

## Materials & methods

### Study cohort

The present cross-sectional study was conducted in the Prince Muteab bin Abdulaziz Hospital (Al-Jouf region, Saudi Arabia) and involved recruiting asthma patients and healthy volunteers in the period from March to September 2020. Physician-diagnosed adult patients with asthma were invited to the study through the E-Health system of the respiratory clinic department at Prince Muteab bin Abdulaziz Hospital, and prior informed consent was obtained through use of the valid questionnaire obtained from the European Community Respiratory Heath Survey (ECRHS.org). The study was approved by the Practical Research Ethics Committee Central IRB log No: 20-32M.

This study included a total of 100 physician-diagnosed asthma participants according to the Global Initiative for Asthma (GINA) criteria. Further, for healthy volunteers as controls, a total of 100 age-matched participants from the Al-Jouf region were included.

### Laboratory investigations

Venous blood samples were collected from all the study participants in tubes with different anti-coagulants for measuring different biomarkers. Each sample in the K2 EDTA tube was subjected to hematology analysis involving a complete blood count (CBC) test, while blood from the trisodium citrate tube was subjected towards evaluation of coagulation parameters. These included prothrombin time (PT), activated partial thromboplastin time (APTT), international normalized ratio (INR), and d-dimer. Another trisodium citrate tube sample was subjected to erythrocyte sedimentation rate (ESR) evaluation.

The CBC test was performed in a CELL-DYNRUBY-YOM2008 hematology analyzer (Abbott Laboratories, North Chicago, IL, USA), while the VIDAS machine Biomerieux Vidas D-Dimer Exclusion II (Biomerieux, USA) was used for d-dimer estimation. The STAGO Fully Automated, Moderate-Throughput Hemostasis Analyzer (The STA Compact Max, USA) was used for measuring PT, APTT, and INR, while ESR estimation was conducted through the Westergren method. The immune cell ratio involving the ENR, NER, and NLR was calculated by standard calculation programs.

### Statistical analysis

Data analysis was performed *via* the Statistical Package of Social Science Software program, version 25 (IBM SPSS Statistics for Windows, Version 25.0. Armonk, NY: IBM Corp.). The Kolmogorov–Smirnova test identified the data to follow a standard normal distribution, as the P value was found to be more than 0.05 for all the variables. An independent sample t-test was used to compare the case-control data and also to compare the smoking and medication status among the patients with asthma. Correlation between the immune cell ratios and coagulation markers between the healthy controls and asthma patients was achieved using the Pearson correlation test. Multivariate linear regression models were performed to explore the predictors of the association between the immune cell ratio, coagulation markers, and hematological parameters between the cases and controls.

## Results

### Study cohort

The current case-control study involved 100 physician-diagnosed asthma patients from Saudi Arabia and an equal number of healthy controls. The mean age for the healthy controls was found to be 31.73 ± 5.51 years and 32.90 ± 5.882 years for the cases. Smokers were only a part of the asthma case group at a frequency of 46%. Furthermore, passive smoking was found at a frequency of 68% among non-smokers, while 48% of the asthma cases reported being exposed to second-hand smoke through smoking parents in childhood.

Among the asthma case group, the mean age of the first attack was noted to be 20.38 ± 8.98 years. Further, 80% of the study participants reported feeling tightness of the chest upon waking up, while 36% reported incidence of wheezing without cold. When considering seasonal variation, the majority of the participants, at 62%, reported attack occurrence in the winter, followed by 18% during the winter and autumn seasons. The mean average of asthma attacks in the past 12 months before the study period was found to be 8.79 ± 0.14. Furthermore, during the study period, 78% of the patients were under medication.

### Hematological and coagulation indices

#### Immune cell scores

Comparison of immune cell counts between the asthma patients and controls found levels of lymphocytes (LYM) and eosinophils (EOS) to be higher among the cases as compared to the controls with statistical significance (LYM—2.91 ± 0.95 vs. 2.4 ± 0.76; EOS—0.4 ± 0.32 vs. 0.16 ± 0.09, p<0.001).

#### Immune cell ratio

In the immune cell ratio comparison, the ENR was found to be higher among the asthma cases as compared to the controls (0.116 ± 0.102 vs. 0.044 ± 0.032, p<0.001). Furthermore, both NER and NLR were lower among the asthma cases as compared to the controls (NER—23.53 ± 45.27 vs. 47.97 ± 65.65, p = 0.042; NLR—1.47 ± 0.7 vs. 1.95 ± 1.1, p<0.001).

#### Coagulation markers

The levels of d-dimer were found to be significantly higher among the asthma cases as compared to the controls (292.07 ± 98.49 vs. 213.44 ± 87.19, p<0.001), while no significant difference was found between the levels of PT, PTT, and INR between the study groups.

#### Hematology parameters

No significant difference in the levels of red blood cells (RBCs), platelets (PLT), hemoglobin (HGB), and ESR was found between the study groups.

### Association analysis between immune cell ratios and hematological and coagulation markers

Among the patients with asthma, a statistically significant and strong correlation was found between the ENR and d-dimer levels (p<0.001). Furthermore, each unit increase in d-dimer was linked with an increase in the ENR of 0.0006 in the patients with asthma. In addition, strong associations between the NER and levels of d-dimer, PT, and INR were also found (p=0.004, p=0.021, and p=0.021, respectively). The association between the ENR and levels of d-dimer was also found among the healthy controls at p = 0.015, and with each unit increase in d-dimer, the ENR was found to decrease by about 0.000094. The association of the NER against the seven factors (d-dimer, PT, PTT, INR, platelet, ESR, and HGB) was not found to be significant in the healthy controls group. In contrast, association of the NER with the seven factors was found at varying levels of significance across the case group. Among the cases, association was also found between the NLR and HGB at p=0.075. The results have been highlighted in **Table 1**.

**Table 1:** Association between immune cell ratio, coagulation markers, and hematological parameters.

	Immune Cell Ratio	Hematology and Coagulation markers	β	95% Confidence Interval for β	P value	Partial Correlation	R^2^
				LL – UL			
Healthy controls	ENR	Intercept	0.098	-0.002 … 0.198	0.054		0.096
		D-dimer	-9.4E-05	-1.7E-04 … -1.9E-05	0.015	-0.208	
		PT	-0.004	-0.019 … 0.011	0.585	-0.055	
		PTT	-4.3E-04	-2.5E-03 … 1.6E-03	0.682	-0.116	
		INR	0.018	-0.148 … 0.185	0.828	-0.017	
		PLT	5.6E-05	-6.2E-05 … 1.7E-04	0.349	0.029	
		ESR	1.7E-04	-9.3E-04 … 1.3E-03	0.764	-0.042	
		HGB	4.0E-04	-2.8E-03 … 3.6E-03	0.805	0.020	
Patients with asthma	ENR	Intercept	-0.057	-0.346 … 0.231	0.693		0.361
		D-dimer	6.0E-04	4.2E-04… 7.7E-04	<0.001	0.568	
		PT	0.008	-0.039 … 0.054	0.738	0.039	
		PTT	0.002	-0.002 … 0.007	0.334	0.113	
		INR	-0.135	-0.601 … 0.332	0.568	0.039	
		PLT	8.8E-05	-2.1E-04 … 3.9E-04	0.561	0.037	
		ESR	4.4E-04	-2.4E-03… 3.3E-03	0.762	-0.002	
		HGB	-0.005	-0.016 … 0.005	0.328	-0.007	
Healthy controls	NER	Intercept	128.647	-69.709 … 327.004	0.201		0.131
		D-dimer	0.070	-0.079 … 0.22	0.352	-0.030	
		PT	7.540	-22.334 … 37.414	0.617	0.079	
		PTT	-2.990	-7.074 … 1.094	0.149	-0.149	
		INR	43.670	-287.021 … 374.361	0.794	0.083	
		PLT	-0.232	-0.466 … 0.002	0.052	-0.117	
		ESR	-1.631	-3.82 … 0.557	0.142	-0.139	
		HGB	-5.063	-11.431 … 1.304	0.118	-0.290	
Patients with asthma	NER	Intercept	179.535	32.781 … 326.289	0.017		0.164
		D-dimer	-0.132	-0.22 … -0.043	0.004	-0.219	
		PT	-28.155	-51.88 … -4.431	0.021	-0.214	
		PTT	-0.045	-2.349 … 2.259	0.969	-0.063	
		INR	265.999	28.276 … 503.721	0.029	-0.175	
		PLT	-0.050	-0.203 … 0.103	0.515	0.011	
		ESR	-0.465	-1.923 … 0.994	0.528	-0.045	
		HGB	1.048	-4.319 … 6.415	0.699	-0.046	
Healthy controls	NLR	Intercept	-0.538	-3.959 … 2.883	0.755		0.082
		D-dimer	0.002	-0.001 … 0.005	0.134	0.047	
		PT	-0.099	-0.614 … 0.417	0.705	0.032	
		PTT	-0.007	-0.078 … 0.063	0.838	0.031	
		INR	2.584	-3.12 … 8.287	0.371	0.063	
		PLT	-0.001	-0.005 … 0.003	0.611	0.049	
		ESR	-0.006	-0.044 … 0.032	0.744	0.047	
		HGB	0.095	-0.015 … 0.205	0.089	0.306	
Patients with asthma	NLR	Intercept	1.016	-1.332 … 3.364	0.393		0.118
		D-dimer	-0.001	-0.002 … 0.001	0.403	0.276	
		PT	-0.096	-0.475 … 0.284	0.618	0.254	
		PTT	-0.019	-0.056 … 0.018	0.311	0.057	
		INR	1.791	-2.012 … 5.595	0.352	0.249	
		PLT	-0.001	-0.004 … 0.001	0.362	-0.150	
		ESR	-0.007	-0.031 … 0.016	0.543	-0.027	
		HGB	0.078	-0.008 … 0.164	0.075	0.200	

ESR, Erythrocyte sedimentation rate, PT, Prothrombin time; PTT, Partial thromboplastin time; INR, International normalized ratio; D-dimer; ENR, Eosinophil-to-neutrophil ratio; NER, Neutrophil-to-eosinophil ratio; NLR, Neutrophil-to-lymphocyte ratio; PLT, Platelets.

*P value was calculated using a multiple linear regression test to find the relationship between the immune cell ratio, coagulation markers, and hematological parameters. P value < 0.05 was considered as statistically significant.

β- a beta level, that is probability of accepting the null hypothesis when it’s false. LL, Lower limit; UL, Upper Limit.

### Association analysis between immune cell scores and hematological and coagulation parameters

Among the asthma cases, levels of EOS were found to correlate with d-dimer at p<0.001. In the case of healthy controls, significant association was found between white blood cells (WBCs) and PLT at p = 0.008, LYM and PLT at p = 0.007, EOS and PLT at p = 0.006, and neutrophils (NEU) and HGB at p = 0.041. The results have been summarized in **Table 2**.

**Table 2:** Correlations between immune cells, coagulation markers, and hematological parameters.

	Markers	ESR		D-Dimer		PT		PTT		INR		HGB		PLT	
		*Corr.	P value	*Corr.	P value	*Corr.	P value	*Corr.	P value	*Corr.	P value	*Corr.	P value	*Corr.	P value
**Healthy controls**	**WBC**	0.044	0.660	0.061	0.550	-0.047	0.640	0.190	0.054	-0.035	0.730	0.170	0.082	0.260	0.008
**Patients with asthma**	**WBC**	-0.140	0.150	-0.009	0.930	0.045	0.660	0.051	0.610	0.019	0.850	0.290	0.004	0.20	0.005
**Healthy controls**	**NEU**	0.038	0.710	0.120	0.240	0.032	0.750	0.180	0.080	0.047	0.640	0.210	0.041	0.170	0.080
**Patients with asthma**	**NEU**	-0.180	0.077	-0.140	0.170	0.048	0.640	-0.006	0.950	0.033	0.740	0.280	0.050	0.047	0.640
**Healthy controls**	**LYM**	-0.028	0.780	-0.073	0.470	-0.140	0.150	0.120	0.250	-0.150	0.104	-0.029	0.770	0.270	0.007
**Patients with asthma**	**LYM**	-0.082	0.420	-0.063	0.530	- 0.058	0.570	0.071	0.480	-0.080	0.430	0.024	0.810	0.31	0.002
**Healthy controls**	**EOS**	0.097	0.340	-0.210	0.033	-0.047	0.640	0.130	0.210	-0.007	0.940	0.120	0.220	0.270	0.006
**Patients with asthma**	**EOS**	-0.004	0.970	0.570	<0.001	0.048	0.630	0.130	0.200	0.031	0.760	0.041	0.680	0.10	0.310

ESR, Erythrocyte sedimentation rate; PT, Prothrombin timel PTT, Partial thromboplastin time; INR, International normalized ratio; D-dimer, WBC, White blood cells; LYM, Lymphocyte; EOS, Eosinophil; NEU, Neutrophil; ENR, Eosinophil-to-neutrophil ratio; NER, Neutrophil-to-eosinophil ratio; NLR, Neutrophil-to-lymphocyte ratio; HGB, Hemoglobin; PLT, Platelets.

Corr.: Correlation.

*P value was calculated using Pearson correlation test to find the correlation between the immune cells, coagulation markers, and hematological parameters in the healthy controls and patients with asthma.

P value < 0.05 was considered as statistically significant.

β- a beta level, that is probability of accepting the null hypothesis when it’s false. LL, Lower limit; UL, Upper Limit.

### Associations between hematological and coagulation markers in smokers and non-smokers

Among the asthma patients, individuals with smoking history were found to have lower levels of LYM in comparison to the patients with no history (2.58 ± 0.77 vs. 3.18 ± 1.01, p <0.001). Apart from the immune cell score, levels of d-dimer were also significantly different between the smoker and non-smoker patients (325313.57 ± 84.39 vs. 273.74 ± 106.43, p = 0.040).

### Associations between coagulation abnormalities and asthma medication status

There was no significant difference between the measured coagulation abnormalities scores (including d-dimer, PT, PTT, INR, and PLT) among the patients who had and did not have a history of use of asthma medications.

### Associations between disease severity and hematological and coagulation markers

Our analysis found a statistically significant association between the levels of d-dimer and asthma attacks at p<0.001, indicating the severity of the attacks to be impacted by d-dimer. Furthermore, a significant association was also found between the levels of PTT and mild persistent asthma attacks at p=0.02. The results have been summarized in **Table 3**.

**Table 3:** Associations between asthma status, coagulation markers, and hematological markers in patients with asthma.

Regression	ESR		D-Dimer		PT		PTT		INR	
	β	P-value	β	P-value	β	P-value	β	P-value	β	P-value
Asthma attack:										
**Mild persistent** **(More than 12 attacks)**	0.450	0.760	86.610	<0.001	0.690	0.200	2.580	0.020	0.080	0.130
**Mild intermittent** **(Between 10-12 attacks)**	-0.690	0.760	140.390	<0.001	0.270	0.730	0.570	0.730	0.020	0.820
History of parental asthma:										
**NO**	-9.460	0.120	104.340	0.300	-1.190	0.580	-1.270	0.790	-0.100	0.640
**YES**	-8.140	0.200	127.570	0.230	-0.890	0.700	-1.510	0.760	-0.084	0.710

ESR, Erythrocyte sedimentation rate; PT, Prothrombin time; PTT, Partial thromboplastin time; INR, International normalized ratio; D-dimer.

*P value was calculated using a multiple linear regression test to find the association between asthma attack and history of parental asthma and coagulation markers and hematological markers.

P value < 0.05 was considered as statistically significant.

β- a beta level, that is probability of accepting the null hypothesis when it’s false. LL, Lower limit; UL, Upper Limit.

## Discussion

The main focus of the current study was to assess the strength of the associations between immune cell ratios and coagulation markers in patients with asthma compared with healthy individuals. The exact role of these immune cells in asthma is not fully understood. However, it is thought that they play a role in the inflammation and narrowing of the airways that are characteristic of asthma. This study found no observable differences between the patients with asthma and healthy controls when comparing the WBC and NEU count. However, the EOS count in the patients with asthma was found to be higher than in the healthy controls. Eosinophils have differential modulations in asthma patients, and many murine models have confirmed evidence from recent studies (18).

In the case of the immune cell ratio, our study found the NLR to be high among the healthy controls as compared to the asthma cases. This finding stands in line with many previous publications, including a recent one by Bedolla-Barajas et al. (2022_ (19). This study compared the concentration of WBCs and the immune cell ratio among asthma patients and a healthy control group and found that the NLR did not differ between the study groups. However, the other groups of immune cell ratios, including the eosinophil–lymphocyte ratio (ELR), ENR, eosinophil–monocyte ratio (EMR), and platelet–lymphocyte ratio (PLR), were found to be high among the cases. The study by Gungen and Aydemir (2017) (20), involving 142 asthma patients and 104 healthy controls, identified the mean NLR to be higher among the cases as compared to the controls (2.2 ± 1.2 vs. 1.83 ± 1.02; p=0.005). Though the association of the NLR with disease severity and hospitalization has been documented across different conditions including cardiovascular disease, kidney disease, and chronic obstructive pulmonary disease (COPD), its association with asthma continues to be contradictory (21, 22). Studies have assessed the factors that impact the heritability of immune cell ratios such as the PLR and NLR. One such study by Lin D. et al. (2016) (5) found heritability to be influenced by age, gender, and environmental factors including seasonal variations and lifestyle. Another cross-sectional study identified the ELR and ENR to be elevated in eosinophilic asthma, while the NLR was found to be elevated in neutrophilic asthma, although blood neutrophil parameters were found to be poor surrogates for levels of sputum neutrophils (22).

Studies have also investigated the role of EOS in asthma, finding that it is crucial for airway hyperresponsiveness and mucus accumulation. Eosinophils are frequently discovered in the airways of those who have asthma. They may contribute to airway swelling and constriction, which may cause asthma symptoms. Furthermore, it was also noted that EOS is crucial for airway remodeling and plays a crucial role in the natural exacerbation of the disease (23). In our study, the ENR was found to be higher among the asthma cases as compared to the controls (0.116 ± 0.102 vs. 0.044 ± 0.032, p<0.001), which is in line with many other publications. Studies have also found that patients with EOS counts of <90 cells/μl are unlikely to develop airway eosinophilia, while patients with counts of >400 cells/μl are expected to develop significant sputum eosinophils (24). The positive impact of eosinophilia has also been demonstrated in recent studies that evaluated the association between asthma diagnosis and endotype and clinical outcomes among patients diagnosed as having coronavirus disease 2019 (COVID-19) infection. This study, involving 10,523 COVID-19 patients, identified patients with asthma to have a lower mortality (adjusted odds ratio [OR], 0.64 (0.53-0.77); P <.001) and lower rate of hospitalization and intensive care unit admission (OR, 0.43 (0.28-0.64); P <.001 and OR, 0.51 (0.41-0.64); P <.001, respectively). Furthermore, patients with blood EOS >/= 200 cells/μL, both with and without asthma, had lower mortality. This indicates eosinophilia, with or without asthma, may be associated with a reduced mortality risk (25). Another study involving COVID-19 patients identified eosinopenia to be associated with a higher risk of intensive care unit admission (OR:2.21; 95%CI:1.42-3.45; p<0.001) (26).

In the case of the impact of d-dimer on asthma exacerbation, our study found a significant association between levels of d-dimer and asthma attacks at p<0.001, indicating the severity of the attacks to be impacted by d-dimer.

D-dimer levels can be elevated during an exacerbation. This is thought to be due to the inflammation that occurs in the airways during an exacerbation. The inflammation can trigger the release of clotting factors, which can lead to the formation of blood clots in the airways (27).

Furthermore, a significant and strong correlation was found between the ENR and d-dimer levels (p<0.001). Studies have aimed to determine systemic blood coagulation during asthma exacerbation compared with the stable state in children aged 5–15 years. This study found d -dimer to be positively correlated with the asthma exacerbation score (R = 0.466, p = 0.027) (28). Studies have also assessed prothrombotic state in asthmatic patients with different disease severity, and the effect of inhaled corticosteroids (ICS). The study found moderate-to-severe asthmatics to have higher d-dimer levels in comparison to mild asthmatic patients and the control group [310.0 (260–1280), 187.5 (160–220), 40.0 (30–70) µg/ml, respectively]. Furthermore, d-dimer levels were also found to be significantly increased among asthmatics using medium-to-high doses of ICS (29).

When comparing smokers and non-smokers in the asthma case cohort, levels of d-dimer were found to be significantly higher among smokers in the present study. Studies have found levels of d-dimer to be higher among current smokers than ex-smokers, who have been shown to exhibit levels higher than non-smokers (30, 31). Further, our study found levels of LYM to be lower among smokers than non-smokers. This finding is also in line with a study by Semenzato U. et al. (2021) (32), who investigated the relationship between blood lymphocyte and its possible decline over time with long-term outcomes in smokers with and without chronic obstructive pulmonary disease. The study found the lymphocyte count to be lower in these patients (1,880 cells/µL) and a decline in the count to be related to the worst outcome among smokers with or without the disease.

The current study found an increase in the number of asthma attacks to be a significant factor that impacted the levels of d-dimer and PTT. Both among mild persistent and intermittent asthma attack cases, the levels of d-dimer were significantly higher, while levels of PTT were increased only in cases of mild persistent asthma attacks. Studies have documented increasing evidence around the involvement of locally derived factors in the activation of the extrinsic coagulation cascade in the asthmatic airway. They have also tested the influence of ICS and plasma exudation in favoring fibrin formation in an asthmatic airway. This study found ICS weaning to increase the levels of plasminogen, thus highlighting that in untreated moderate asthma, increased risk of fibrinolysis is corrected by ICS. However, severe asthma and ensuing high-dose corticosteroid therapy were found to be associated with the profibrinogenic and antifibrinolytic environment in the airway (33). Studies have also documented the activation of coagulation to be induced by leakage of plasma proteins in the bronchoalveolar space, including the lung resident tissue factor, which is the key mediator of coagulation that initiates the coagulation and thrombin that further transforms fibrinogen to fibrin (34). Our findings also highlight a significant association between the immune cell ratio and coagulation abnormality, which is the ENR and d-dimer. These findings are also in line with previous publication reports that identified oral corticosteroid therapy inducing neutrophil recruitment in the airway of mild asthma patients (35). Studies have also highlighted the common occurrence of airway neutrophilic inflammation in refractory asthma and among patients with persistent airway obstruction (PAO). Furthermore, eosinophilic inflammation was also found to be predominant in the non-PAO cohort (36).

The possible limitations that may have affected the results are discussed here. The study cohort consisted of a relatively small number of participants, which may limit the generalizability of the findings. A larger sample size could provide more robust results and a better representation of the population. The study included only physician-diagnosed asthma patients from Saudi Arabia, which may introduce selection bias. The results may not be applicable to other populations or individuals with undiagnosed asthma. Future studies should aim to include a more diverse and representative sample. The data collected in this study relied on self-reported information from the participants, which may be subject to recall bias. The participants may not have accurately recalled or reported their exposure to certain risk factors or symptoms. This could potentially impact the accuracy of the associations observed in the study. The study utilized a cross-sectional design, which can only establish associations and not causal relationships. Longitudinal studies or randomized controlled trials would be needed to establish a cause-and-effect relationship between the immune cell ratios, hematological parameters, and coagulation markers in asthma patients. Despite efforts to control for confounding variables, there may still be unmeasured or residual confounders that could have influenced the observed associations. Factors such as socioeconomic status, comorbidities, and medication use were not fully accounted for in this study, and they could potentially impact the results.

## Conclusion

**Figure 1:**
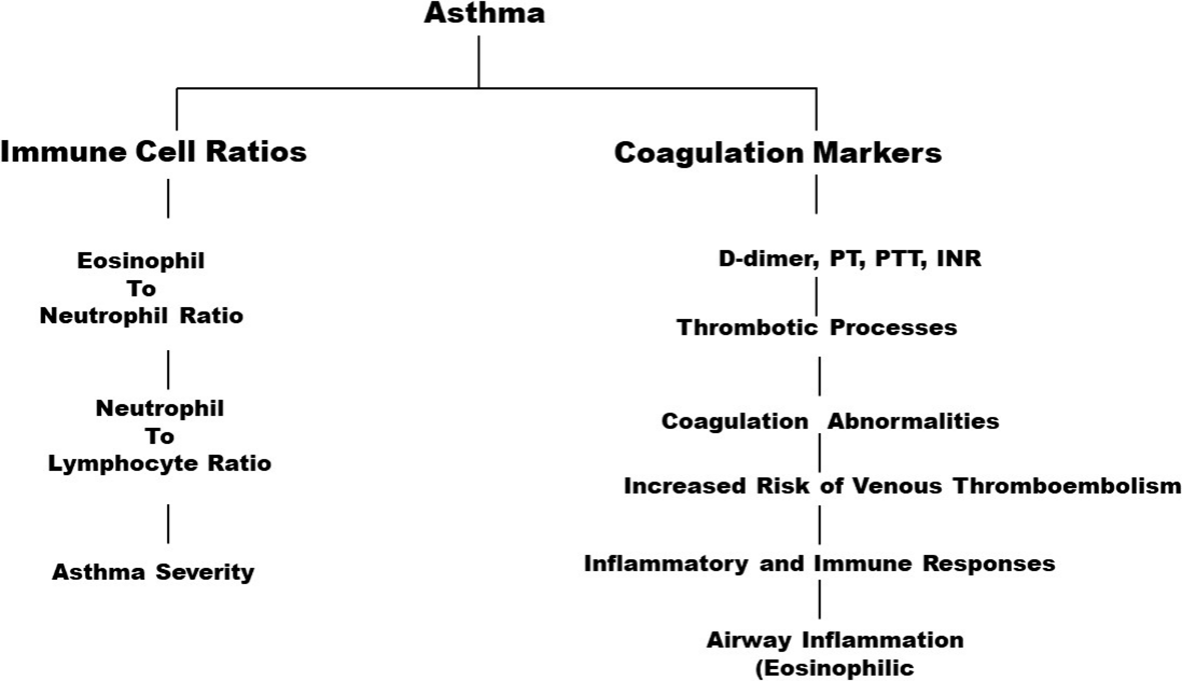
Significant associations between immune cell and coagulation markers in asthma.

The present study highlights significant associations between the immune cell ratio ENR and d-dimer levels between asthma patients and controls (**Figure 1**). The strength of the study lies in the fact that it investigates multiple factors to predict the occurrence of blood thrombosis in patients with asthma. Based on the findings of this study, it can be further recommended that hospitals do include studying factors such as ENR, NER, and NLR in the treatment of asthma to develop the best treatment module. The study also implies that medical practitioners should take coagulation markers into consideration while addressing patients with asthma as these indicators can provide the means for determining the best form of treatment.

## Data availability statement

The original contributions presented in the study are included in the article/supplementary material. Further inquiries can be directed to the corresponding authors.

## Ethics statement

The study was approved by the Practical Research Ethics Committee Central IRB log No: 20-32M. Written informed consent to participate in this study was provided by the participants’ legal guardian/next of kin.

## Author contributions

FA, ZA, RS, and JJ: Concepts, design, data analysis, statistical analysis, manuscript preparation, manuscript review, and guarantors. SA, AA, AM, and RS: Definition of intellectual content, literature search, experimental studies, data acquisition, and manuscript editing. All authors contributed to the article and approved the submitted version.
